# Computational Analysis of Axonal Transport: A Novel Assessment of Neurotoxicity, Neuronal Development and Functions

**DOI:** 10.3390/ijms13033414

**Published:** 2012-03-12

**Authors:** Yoshio Goshima, Tomonobu Hida, Toshiyuki Gotoh

**Affiliations:** 1 Department of Molecular Pharmacology and Neurobiology, Yokohama City University, Graduate School of Medicine, Yokohama 236-0004, Japan; E-Mail: tomonobu1105@hotmail.com; 2 Graduate School of Environment and Information Sciences, Yokohama National University, Yokohama 240-8501, Japan

**Keywords:** axonal transport, anti-neoplastic agents, microtubules, kinesin, dynein

## Abstract

Axonal transport plays a crucial role in neuronal morphogenesis, survival and function. Despite its importance, however, the molecular mechanisms of axonal transport remain mostly unknown because a simple and quantitative assay system for monitoring this cellular process has been lacking. In order to better characterize the mechanisms involved in axonal transport, we formulate a novel computer-assisted monitoring system of axonal transport. Potential uses of this system and implications for future studies will be discussed.

## 1. Introduction

Neurons are highly polarized cells having axons and dendrites, with proper neuronal differentiation, survival and function heavily dependent on the axonal and dendritic transport machinery [1,2]. In axons, the molecules required for synapse formation are synthesized in the cell body and transported to the synaptic area. Molecules such as neurotrophins are received in the growth cone or nerve terminal, and transported to the cell body. This transport machinery depends on two families of motor proteins to transport molecules along microtubules, kinesin and dynein [1,3]. Defects in axonal transport reportedly can lead to neurodegenerative pathology and related disorders, such as Schizophrenia, Alzheimer’s (AD), Parkinson’s, other motor neuron diseases and drug-induced neuropathies [4–6], highlighting the importance of understanding the mechanisms of axonal transport.

For the analysis of axonal transport, conventional assay systems have been utilized, such as isotope tracer techniques, video-enhanced differential interference contrast microscopy or fluorescence recovery after photobleaching (FRAP) via the introduction of green fluorescence protein (GFP)-tagged proteins [7]. Current studies suggest that regulation of axonal transport occurs at multiple levels, including modulation of the microtubule track, cargo-specific adaptors and scaffolding proteins that coordinate cargo-bound motors [8]. Oddly enough, little is known about the regulatory mechanisms of axonal transport. This may partly be because earlier methods are time-consuming and depend on a subjective element. Indeed, up to now, little attention has been paid at ligand-specific regulation of axonal transport. We previously reported using a video-enhanced contrast differential interference video camera system to establish that a repulsive axon guidance molecule, Semaphorin3A (Sema3A), induces anterograde and retrograde axonal transport [9–11]. It is only recently that we found that Sema3A plays an important role in neuronal morphogenesis, and functions by regulating axonal transport [12]. Therefore, the development of a simple and objective assay system to evaluate axonal transport has long been awaited. We have recently developed a computer-assisted monitoring system for axonal transport [4]. In this review, we will just briefly summarize the physiology and pathophysiology of axonal transport, and discuss about the validity and general versatility of this method, and our tasks for the future.

## 2. Physiological Functions of Axonal Transport

### 2.1. The Role of Axonal Transport in Neural Survival

In the peripheral and central neurons, neurotrophins are important regulators for the survival, differentiation and maintenance of nerve cells. Recent studies indicate that axonal transport plays an important role in neuronal survival [13,14]. In fact, prosurvival signals are blocked by the inhibition of retrograde axonal transport [15]. Nerve growth factor (NGF), brain-derived neurotrophic factor (BDNF), neurotrophin-3 and neurotrophin 4/5 are members of the neurotrophin family. Each neurotrophin binds with high affinity to a specific Trk receptor tyrosine kinase. These growth factors can also induce cell death through the p75 neurotrophin receptor, a member of the tumor necrosis factor receptor superfamily [16]. Upon ligand binding, Trk receptors undergo dimerization and autophosphorylation on several key tyrosine residues, which then serve as docking sites for a variety of effector proteins. NGF, which is produced and released by target cells innervated by peripheral neurons, acts on TrkA receptors located on the axon terminals of innervating neurons, initiates biochemical signals locally within distal axons and sends signals retrogradely to neuronal cell bodies and ultimately to their nuclei. Recent findings provide strong support for a “Signaling Endosome Model” in which NGF-TrkA complexes undergo internalization in distal axons and are then physically translocated along the axonal microtubule network to cell bodies where they can initiate or propagate prosurvival signals [17]. However, the identity of the endocytotic vesicular carrier(s) and the mechanisms involved in retrogradely transporting the signaling complexes remain unknown [18].

### 2.2. The Role of Axonal Transport in Neural Network Formation

During development, after postmitotic division, neurons extend their neurites, and recognize their environments to make synapses with their appropriate target cells. Although a role of local protein synthesis in growth cone has now been appreciated in this process, all the proteins required in the axon, growth cone and synaptic terminal must be transported down the axon after they are synthesized in the cell body. In neurons, various kinds of receptors for axon guidance molecules must be localized properly to axons or dendrites for appropriate neuronal wiring. Signaling outcomes downstream of ligand binding are determined by the location, levels and residence times of receptors on the neuronal plasma membrane. Therefore, the mechanisms controlling the trafficking of these receptors are crucial to the proper wiring of circuits. Indeed, the molecular machinery involving membrane trafficking has been implicated in axon guidance.

For example, UNC-51 is a serine/threonine kinase homologous to yeast Atg1, required for autophagy, a form of catabolic vesicle trafficking. In the *unc-51 Caenorbabditis elegans* mutants, some axon guidance defects have been observed [19]. In addition, in *unc-51* worms, an aberrant localization of netrin/UNC-5 and its receptor UNC-6 [20,21], are associated with axon guidance defects. Given that UNC-6 is required for dorso-ventral axon guidance, altered localization of UNC-6 in this mutant probably causes or reflects UNC-6 secretion defects, which should result in dorso-ventral axon guidance defects [20,21]. In support of this hypothesis, *unc-51* mutant shows defective dorsally directed axon pathfinding by DD/VD neurons [20]. Such defects in axon guidance or neurite extension might also be caused by impaired membrane supply at the growth cone via non-selective axonal transport. Surprisingly however, our findings suggest that UNC-51 regulates the localization of synaptobrevin and UNC-5, at least in regard to its accumulation in the cell body, but does not appear to regulate the localization of proteins generally in neurons [20]. More direct evidence for this link between axon guidance processes and axonal transport is seen in the mutant worm *Kif1A/unc-104* [22]. In *unc-104*, the neurons show an abnormal accumulation of Venus::UNC6 in the cell body, and this mutant shows defects in the guidance of dorso-ventral axons, suggesting that the abnormal localization of UNC-6 disturbs the positional information it provides (Asakura *et al.*: unpublished observation). These findings suggest that axonal transport plays an important role in axon guidance mechanisms by regulating the localization of axon guidance molecules or their receptors. Another example that shows close relationship between axonal transport and axon guidance is KIF2A [2]. KIF2A is expressed predominantly in the developing brain, especially in neuronal growth cones. In the brains of *Kif2A* knockout mice, axon bundles labeled with DiI crystals show an increased number of horizontally running neurites in mutant mice, whereas axons run longitudinally in wild-type mice. *In vitro* experimentation shows that KIF2A depolymerizes microtubules in a ATP-dependent manner. Consistently, in the absence of KIF2A, microtubules continue to elongate even after reaching the cell periphery, while in wild-type cells, elongating microtubules began to depolymerize when reaching cell periphery. These observations raise the question of how axonal transport is regulated so that these guidance molecules are presented to growth cones for proper axonal navigation or wiring.

### 2.3. Axonal Transport and Neural Plasticity

Neurotrophic factors such as NGF and BDNF play important roles, not only in neuronal development and survival, but also in neural plasticity. Recently, presynaptic BDNF promotes postsynaptic long-term potentiation (LTP) in the dorsal striatum [23]. On the other hand, as described above, neurotrophic factors activate active downstream effectors such as Trk receptors at presynaptic sites and are transported to the same via a retrograde signaling endosome [8]. Therefore, it is conceivable that neurotrophins regulate neural plasticity through both postsynaptic and presynaptic mechanisms. However, the question remains open as to whether retrograde signaling elicited by neurotrophic factors are involved in neural plasticity.

### 2.4. Pathophysiology of Axonal Transport

Evidence implicating axonal transport defects in the pathogenesis of neurodegeneration has come from the identification of mutations in the motors that drive axonal transport [8]. For example, the identification of mutations in dynein provides evidence that defects in axonal transport are sufficient to cause neuronal degeneration. Furthermore, recent studies suggest that slowing of axonal transport is an early event in the pathogenesis of a number of neurodegenerative diseases such as amyotrophic lateral sclerosis (ALS) [24,25], Huntington’s disease (HD) [26,27] and AD [28]. Although the exact mechanisms of impaired axonal transport are unknown, some suggestive evidence has been obtained in recent years. In AD, the pathological hallmarks, neurofibrillary tangles, neuropil threads and senile plaques, are potentially linked to alterations of the axonal compartment. Familial AD mutations in β-amyloid precursor protein (βAPP) and in presenilin genes alter the production of amyloid-β peptides (Aβs), the major constituents of senile plaques, suggesting that proteolytic processing of βAPP into Aβs plays a central role in AD pathogenesis. Impairing axonal transport by reducing the dosage of a kinesin molecular motor protein enhances the frequency of axonal defects and increased Aβs levels and amyloid deposition [28]. HD is one of nine neurodegenerative diseases that result from the expansion of CAG repeats, leading to proteins containing abnormally long polyQ tracts. It has been proposed that misfolding of the mutant protein triggers a cascade of events, ultimately causing disease. Huntingtin is a cytoplasmic protein with unknown function. In HD brain, aggregates of mutant huntingtin are observed in nuclear inclusions and in dystrophic neurites. Expression of human huntingtin exon 1 with expanded polyQ causes axonal transport defects in larval neurons of *Drosophila*, perhaps by blocking axonal processes by polyQ accumulations, neuronal cell death and neurodegeneration [27]. However, the mechanisms and pathophysiological significance of axonal transport are unknown at present.

### 2.5. Comparison of the Analytical Methods for Axonal Transport

A growing body of evidence for the importance of axonal transport has therefore prompted us to establish a computer-assisted software system to monitor axonal transport. Recently, similar attempts have been reported independently to detect and track vesicles along neuronal axons [4,29,30]. The time-sequence images of moving vesicles, which are observed by fluorescence microscopy with probes, such as fluorescence recovery after FRAP, GFP, and CM-DiI, are used for analyzing axonal transport as inputs. The methods are classified into two approaches: the particle tracking in time-sequence image frames [4,29,30] and analysis using a kymograph image [29–31]. We will briefly introduce these methods in this section.

### 2.6. Difficulties in Axonal Transport Analysis

**Figure 1:**
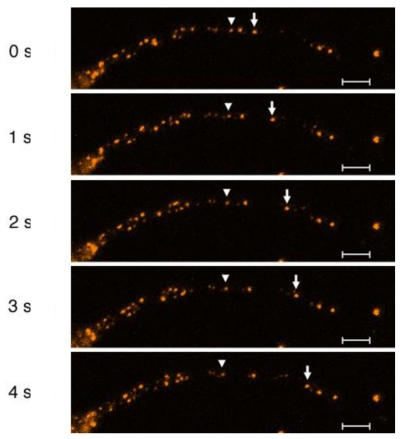
Axonal transport visualized with CM-DiI staining in the axon of chick dorsal root ganglion (DRG) neuron. Dissociated DRG neurons were plated and 12–14 h thereafter CM-DiI was solubilized and added to the cultured neurons. To visualize moving particles, CM-DiI destaining was performed by further incubating the DRG neurons for 6 h in the absence of CM-DiI. The particles indicated by the arrows and arrowheads exhibit representative continuous anterograde and retrograde movement, respectively. From [Goshima *et al.* 2010] [4].

Figure 1 shows frames of a CM-DiI-labeled time sequence image. As shown in this figure, the following difficulties appear in the detection, tracking and analysis of fluorescence microscopic axonal transport images.

1. The gray scale of the vesicle is not similar and the images have a lot of random noise, which made vesicle detection difficult using simple image processing technologies.
2. The vesicles move bi-directionally along the axon with different velocities and/or opposite directions.
3. Since the vesicles move in close proximity in a narrow passageway, the vesicles may appear as a single particle on an image frame and then as several particles on the next.

These difficulties have to be solved when developing methods of axonal transport analysis using fluorescence microscopy. As discussed in the following section, in order to differentiate between those vesicles with different velocities and directions in a narrow passageway, strategies have been explored, such as use of Bayesian estimation [30], particle movement diagram [4] and kymograph image [29,31].

### 2.7. Computer-Based Particle Detection and Tracking Methods

The approach using the particle tracking in time-sequence image frames generally consists of particle detection, tracking and analyzing processes. In the detection process, particles on each frame in the input time-sequence image are firstly detected. In the tracking process, a pattern of each particle detected in an image frame is searched on those in the next frame to make a correspondence between the patterns in successive frames. Then, the traffic paths of particles are also found through the whole image sequences in this process. After finding the traffic paths, quantitative analysis is made, such as the velocity distributions of the antero- and retrograde movement along an axon.

In order to overcome the above-mentioned difficulties on vision-based axonal transport analysis, Broeke *et al.* propose a method for tracking particles which are detected using a heuristic approach on subsequence image frames [30]. In their method, they introduced an appearance model which describes the pixel intensities of a particle as a discrete histogram and a multi-hypothesis tracking which enables the finding of paths, even when particles are observed to merge into one, and, conversely, when one particle appears to split into many. They have also proposed a method using a recursive Bayesian estimation algorithm and auction algorithm to reduce computation time while preserving the accuracy of detection and tracking.

**Figure 2:**
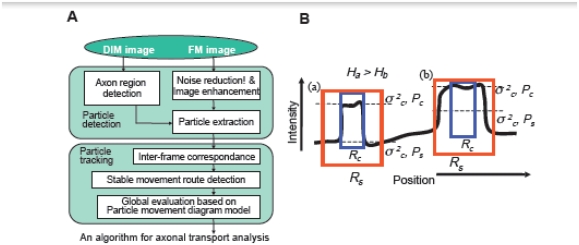

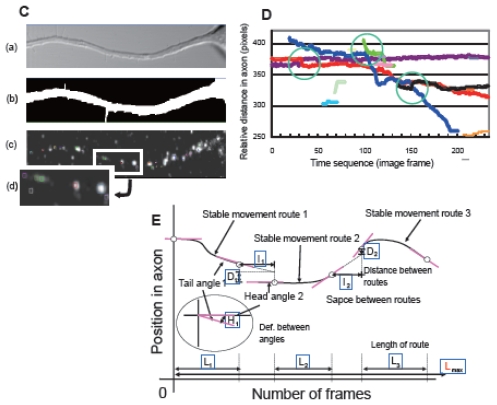
An image-processing algorithm of axonal transport analysis. The proposed algorithm for axonal transport analysis consists of two phases, particle detection and particle tracking (**A**). In panel (**B**), a solid line shows the cross section of an image. Where size of the region fits a vesicle, as in position (a), both of the variances, *σ_c_*^2^ and *σ_s_*^2^, in both center and surround regions become low, and the difference between the mean intensities *P_c_* and *P_s_*, high. On the other hand, where the size does not fit a vesicle, as in position (b), either variance, *σ_c_*^2^ or *σ_s_*^2^, becomes high, and the difference, low. Consequently, the ratios *H_a_* and *H_b_* of variables *of inter- to intra-regions* have a high value at position (a) and a low value at (b). The detected particles are tracked by global evaluation using a *particle movement diagram model* after the determination of successive image frame correspondence. Examples of the results of particle detection (**C**) and of the particle movement diagram (**D**) are shown. In panel (**C**), an input of the differential interference image (a), an image after detection of the axonal region detection (b), and a result of the particle detection on a fluorescence microscopic image frame (c) are shown. A magnified image of the boxed area in image c is shown in image d. In panel (**D**), the lines represent the time course of the movement of each particle in the diagram, which is similar in appearance to a train schedule diagram. The circles in the figure show the crossing points in the diagram, where the particles encounter or get in front of each other; (**E**) Parameters of a global reconstruction evaluation. *Li*, *Ii*, *H*, and *Di* are the time durations of the stable route element *i*, the time interval, the angle, and the distance between the elements *i* and *i* + 1, respectively. From [Goshima *et al.* 2010] [4].

To overcome the difficulties mentioned above, we have taken a different approach. First, we employed chloromethylbenzamide dialkylcarbocyanine (CM-DiI) [4] as a probe to visualize membranous organelles being transported along the axon in chick dorsal root ganglion (DRG) neurons. We have tried various kind of fluorescence dyes, GFP-tagged proteins *etc*. to visualize axonal transport. Among them, we found CM-DiI staining produces the highest signal-to-noise ratio and resolution in live imaging of moving vesicles along the axons. We recorded the moving CM-DiI labeled particles with a laser-scanning microscope, analyzed the axonal transport rate and examined the effects of several pharmaceutical agents on axonal transport. We studied the subcellular distribution of CM-DiI. The signal of CM-DiI was associated with EGFP-tagged lysosomal-associated membrane protein introduced into DRG neurons, suggesting that the majority of the moving particles labeled with CM-DiI are composed of endosomal and lysosomal vesicles in DRG neurons. In our method, the differential interference and fluorescence microscopic sequential images are used as inputs. Our method also consists of two processes of analysis, particle detection and particle tracking (Figure 2A). The software consists of two phases, the detection of a particle and then the tracking of a particle. The software details of this system are described below. In the particle detection process, the axonal region is first detected on an image using a differential interference microscopic image (Figure 2C). Subsequently, noise reduction and particle shape enhancement are carried out using median filtering and convolution filtering on the fluorescence microscopic images. The resulting image revealed CM-DiI–labeled vesicles in the axonal regions. Since the vesicles have a wide range of intensities, general binarizing technologies do not always detect them stably. In order to solve this problem of vesicles having a wide intensity distribution and background having random noises, particles are detected by a method of estimating the shape of particles using separability filtering [32]. Two designated regions, the central region *R_c_* and the surrounding region *R_s_* around a particle, are considered in this method, then the ratio of the variances *H* = σ*_b_*^2^/σ*_i_*^2^ of the inter-region to the intra-region is evaluated. In our particle detection, using the filters with different radius, the size of the filter with maximum output is considered as that of the vesicle. The use of separability filter bank with different sizes enables us to detect both position and radius size of a particle shown in Figure 2B, even for particles of weak intensity with background noise.

In our method, particle tracking is composed of local and global tracking processes. The local tracking process is try to find the correspondence between the patterns of the same vesicle in successive image frames based on the weighted average of differences in the average intensity, the motion vector and the detected particle size on the successive frames. To reduce the tracking errors that may occur using only local tracking, we use a train schedule-like diagram, as shown in Figure 2D. In the diagram, lines represent the time courses of moving particles. We call it a *particle movement diagram*. Using the *particle movement diagram*, it was easy to determine whether a particle tracking route was stable or not: At the crossing points in the diagram, particles encounter or get in front of each other, which may increase the possibility of errors on the results of local tracking. On the other hand, where each particle moved alone on a line between crossing points, the likelihood of error would be low. In the global tracking, the tracking routes are first divided into stable route elements at the crossing points. Then, each combination of the stable route elements is evaluated to find the set of optimum tracking routes, so as to minimize the following evaluation function using a genetic algorithm [33].

$$E_{a}=C_{h}\sum \left|H_{i}\right|+C_{d}\sum \left|D_{i}\right|+C_{i}\sum I_{i}-C_{l}\sum L_{i}$$

where, *H_i_*, *D_i_*, and *I_i_* are the difference between the tail and head angles, the distance and the frames between the stable route element *i* and the successive route element *i* + 1 in a given evaluation route; *L_i_* is the number of frames of the element *i*, as shown in Figure 2E. By minimizing *E_a_*, the elements smoothly connected without gaps and with long paths are found for members in an evaluation route.

Welzel *et al.* proposes a method using Hough transform [31], in order to detect line elements in the image map stably under the difficulties that appeared on the fluorescence microscopic axonal transport images. The Hough transform is a well-known algorithm to extract lines, which has been applied to various fields of image analysis [34], such as automated industrial inspection, computer vision and recognition systems. Their method first detects the axonal region and generates the kymograph from the input time sequence image frames. The binary image is obtained from an edge image after a Canny edge detector is applied to the kymograph. Then, the edge points in a binary kymograph image are voted on a parameter accumulation space. Since the accumulations in the parameter space correspond to lines in the kymograph image, even weak lines can be detected stably on the parameter space by finding the local maxima.

**Figure 3:**
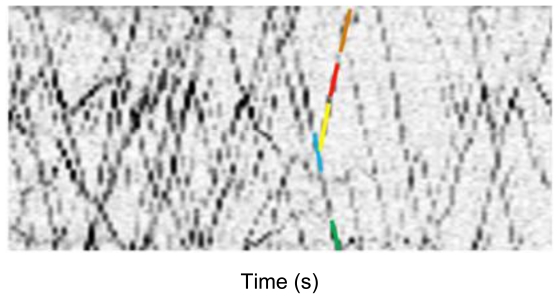
Frames from a video (18 μm × 18 μm region at 1.8 fps) along mouse cortical neurons showing transport of BDNF granules. A space–time map (kymograph) of the video showing the same granule motion. From [Mukherjee *et al.* 2011] [29].

Mukherjee *et al.* proposed another method using kymograph [29]. After generating a kymograph image, they proposed a particular filtering method to sharpening lines on the kymograph, which is based on a voting algorithm defined by orientation distribution function. Then, the lines are detected using an algorithm of minimum set covering, so as to minimize the total cost of curve coverage per unit area on the kymograph image, in order to avoid the above-discussed difficulties of fluorescence microscopic axonal transport images (Figure 3).

### 2.8. Velocity Distribution of Particle Movements

The goal of computer-based methods is generally to obtain the “velocity distribution of particles” of axonal transport from the particle paths obtained from the tracking process, by evaluating the number and instantaneous velocity of particles moving along the axons. However, we found different kinds of “velocity distribution of particles” were reported in previous papers: one is a velocity distribution of all particles in the axonal region, and another is a velocity distribution of passing particles, which are counted at a determined point in an axon. The velocity distribution of passing particles is suitable for representing materials flow, since the distribution tends to become high in proportional to the particle velocity. For example, this distribution of static particles would always be zero. On the other hand, distribution does not depend on their velocity. So, when we need to compare the results from the different distribution, some compensation is needed before comparison.

**Figure 4:**
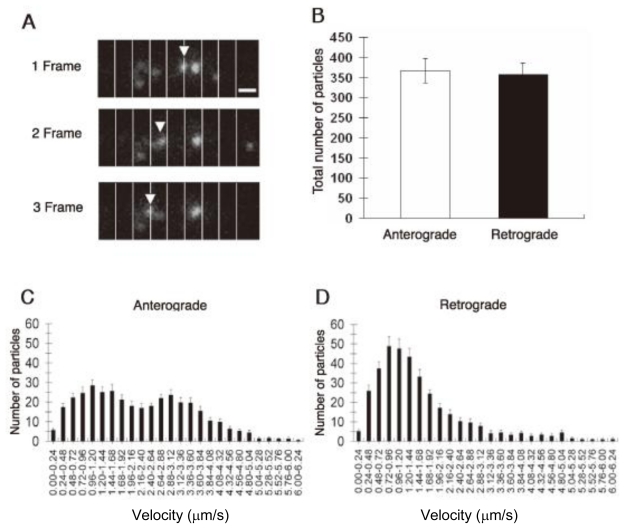
Analysis of axonal transport of particles visualized with CM-DiI in the axon of DRG neurons. (**A**) Images of a moving particle visualized with CM-DiI in an axon. Arrowheads indicate continuous movement of CM-DiI-labeled particles crossing a line drawn every 1 μm on an axon; (**B**) The summation of the number of antero- and retrograde moving particles crossing the 74 virtual lines drawn every 1 μm on the axon was estimated for 2-min time periods (one frame). Values are the means ± S.E.M. of the number of moving particles in each fraction (*n* = 14). Instantaneous velocity histograms of antero- (**C**) and retrograde (**D**) axonal transport are shown. Data are means ± S.E.M. (*n* = 15). The velocity range is 0.24 μm/s. Scale bar, 1 μm. From [Goshima *et al.* 2010] [4].

In our previous report, we estimated and compared the number of CM-DiI-labeled vesicles moving across successive lines drawn every 1 μm from the cell body and perpendicularly to the long axis of the axon (Figure 4A) [4]. We found that the number of anterograde and retrograde vesicles varied among the monitoring points along the axons, consistent observation that the movement of organelles in axonal transport is salutatory, and individual organelles start and stop repeatedly. To monitor movement dynamics of axonal transport, we therefore sought to determine the instantaneous velocity of CM-DiI-labeled vesicles along axons. Furthermore, to minimize data fluctuation, we estimated the summation of the data obtained from a total of more than 70 virtual lines drawn at intervals of 1 μm along the axon starting from the origin of the axon. The histogram of anterograde and retrograde axonal transport monitored with this system revealed a bimodal distribution pattern in anterograde axonal transport, with two peaks being observed at the velocity of 0.96 and 2.88 μm/s (Figure 4C). The histogram of retrograde axonal transport revealed a unimodal pattern, with one peak at the velocity of 0.72 μm/s (Figure 4D). These distribution patterns are consistent with previous observations on the instantaneous velocity of anterograde and retrograde axonal transport [10,35]. On the other hand, Broeke *et al*. proposed a model and a recursive Bayesian estimation algorithm that exploit intrinsic information contained in an image sequence obtained from cultured cortical neurons overexpressing Sema3A-EGFP [30]. An EGFP-tagged version targets large dense core vesicles, similar to endogenous Sema3A, and Sema3A labeled vesicles, showing bi-directional movement along axons. The algorithm is sequential and uses information extracted from previous frames to predict the most likely object configurations. In this system, they evaluated average vesicle velocity of 0.45 μm/s for anterograde, and of 0.67 μm/s for retrograde transport, respectively. Although the value for retrograde axonal transport is comparable to those obtained with CM-DiI-labeled vesicles in DRG neurons [4], the velocity histogram for anterograde axonal transport does not show bimodal distribution, which provides contrast to our results of anterograde transport. The average velocity of anterograde axonal transport of Sema3A labeled vesicles (0.45 μm/s) appears to correspond to the lower value (0.96 μm/s) of instantaneous velocity of DiI-labeled particles. As mentioned above, there are two kinds of the velocity distribution of vesicles: the velocity distribution of all particles in the axonal region, and the velocity distribution of passing vesicles. Although the exact reasons are unknown, it is possible that these two different kinds of velocity distribution may be related to this discrepancy on the velocity histogram of anterograde axonal transport [4,30].

### 2.9. An Example of Computational Analysis of Axonal Transport: A Neurotoxicity Test

Various kinds of therapeutic agents have neurological side effects, and some have been associated with the impairment of axonal transport [5,27]. However, there have been no reports of systemic drug screening based on the effects on axonal transport. We investigated the effects of various pharmacological reagents on axonal transport and checked whether our automated analysis system was applicable in this regard: this comprised an important proof-of-principle required for the further development of drug screening. We first tested the effects of lidocaine on axonal transport in DRG neurons. Lidocaine is widely used as a local anesthetic, and has been reported to exert an inhibitory effect on axonal transport [36]. Lidocaine (10 mM) decreases the number and the velocity of the CM-DiI-labeled vesicles. The lower concentrations of lidocaine (0.1 and 1 mM) produced no effect (data not shown). Interestingly, lidocaine preferentially suppressed the antero- and retrograde moving particles with a velocity of less than 2.16 μm/s.

Many anti-cancer drugs have neuropathic side effects. For example, paclitaxel stimulates and vincristine inhibits the polymerization of β-tubulin to α-tubulin, resulting in the appearance of microtubule bundles and some aberrant structures in the mitotic phase of the cell cycle [37]. A possible disturbance of microtubule functions may affect axonal transport and other cellular functions of sensory neurons. However, the mechanisms for untoward effects, including neuropathic side effects, are largely unknown. If neuropathic side effects are associated with the disturbances of axonal transport, anti-neoplastic agents without neuropathic side effects may have no effect on axonal transport. Using our system, we investigated the effects on axonal transport of several anti-cancer drugs having different sites of action. We treated DRG neurons with paclitaxel, vincristine, cisplatin, oxaliplatin, methotrexate and 5-fluorouracil. Cisplatin and oxaliplatin react with DNA, forming both intrastrand and interstrand cross-links. Cisplatin also inhibits the polymerization of microtubules. Methotrexate (an inhibitor of enzyme dihydrofolate reductase) and 5-fluorouracil (an inhibitor of tymidine monophosphate) inhibit DNA synthesis. As expected, we found that paclitaxel, vincristine, cisplatin and oxaliplatin, all disrupt both antero- and retrograde axonal transport and decrease the number of CM-DiI-labeled vesicles. Furthermore, this system also allows us to confirm concentration-dependent inhibition by paclitaxel (1, 10 and 100 nM), vincristine (10, 50 and 100 pM) and cisplatin (0.1, 1 and 10 μM) of antero- and retrograde axonal transport. The IC_50_ values for antero- or retrograde axonal transport of paclitaxel, vincristine and cisplatin were 4.1 or 5.5 nM, 100 or 130 pM, and 3.0 or 5.5 μM, respectively. These values are comparable to the effective concentrations that inhibit the growth of tumor cells [38–40], further indicating the validity of this assay system for axonal transport. These findings together suggest that neuropathic side effects are associated with the disturbances of axonal transport.

**Figure 5:**
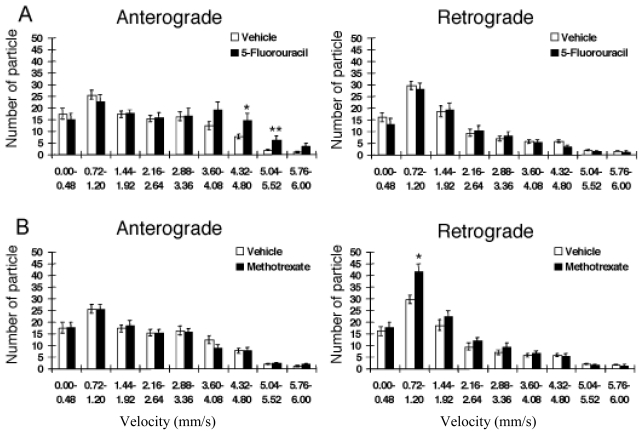
The effects of anti-neoplastic agents, 5-fluorouracil (100 μM) (**A**), methotrexate (100 μM) (**B**) on the number of moving particles (/2 min) labeled with CM-DiI in axons of DRG neurons 24 h after the application of anti-neoplastic agents. Data are means ± S.E.M. (*n* = 5). * *p* < 0.05, ** *p* < 0.01 (Mann-Whitney’s *U*-test), compared to corresponding control. From [Goshima *et al.* 2010] [4].

Using this system, we have discovered that the effects of these pharmacological agents have differential effects on each component of axonal transport. Oxaliplatin shows a tendency to inhibit the fast component of antero- and retrograde axonal transport with a velocity faster than 2 μm/s (Figure 5). On the other hand, paclitaxel inhibits moving vesicles with a velocity slower than 3 μm/s. We also found that 5-fluorouracil and methotrexate do not produce significant effects on axonal transport, but increase the fast and the slow components of antero- and retrograde axonal transport, respectively (Figure 5). In this case, neither 5-fluorouracil nor methotrexate suppressed the total number of moving particles (data not shown). It is unknown how these agents augment the slow components of axonal transport. It would be interesting to note the difference between 5-fluorouracil and paclitaxel or vincristine. Intracellular cargos are transported by multiple motor proteins, and each motor has a characteristic velocity. These agents may have distinct sites of action, which may have relevance to their differential effects on axonal transport.

## 3. Experimental Section

CM-DiI was purchased from Invitrogen (Eugene, Oregon, USA). Poly-L-lysine, sodium azide, nocodazole, vinblastine sulfate, cisplatin, oxaliplatin, 5-fluorouracil, cytochalasin B and lidocaine were from Sigma (St. Louis, MO, USA). Paclitaxel was from Wako (Osaka, Japan). Methotrexate was from Fluka (Buchs, Switzerland). DRGs were removed from embryonic day-7 chick embryos, and were treated with 0.25% trypsin in Ca^2+^- and Mg^2+^-free phosphate buffered saline at 37 °C for 6 min, and stopped by adding trypsin-free medium, as described [41]. The cells were washed three times in Ham’s F12 medium (Sigma) containing 10% fetal bovine serum and 2.5 S nerve growth factor (10 ng/mL; Wako), dissociated in the medium, and seeded onto a 35-mm cell culture dish (CORNING, USA). The cells were cultured for 1 h, the medium was collected, and replated onto a Poly-l-lysine-coated 35-mm glass base dish (Iwaki, Japan). The cells were then cultured at 37 °C for 12–14 h. The cultured cells were washed three times in Leibovitz’s L-15 medium (L-15 medium) (GIBCO, Eggenstein, Germany), and cultured for 30 min in L-15 medium containing CM-DiI (2 mM) and 2.5 S nerve growth factor (10 ng/mL; Wako). The cells were washed three times with L-15 medium, then incubated for 6 h in the CM-DiI-free culture medium to remove any excess dye loaded in the DRG neurons. The absolute value of the number of DiI-labeled moving particles (/2 min) at the start of the experiments varied among the DRG neurons examined. This was probably due to certain differences in the culture conditions and/or developmental stages of the neurons. To characterize the particles stained with CM-DiI, double staining with CM-DiI and mitoTracker or erTracker was performed in DRG neurons. The procedures involving the experimental animal complied with the animal care guidelines of the National Institutes of Health and the animal ethics committee of Yokohama City University.

CM-DiI-labeled moving particles in the axons of cultured DRG neurons were observed using a laser-scanning microscope with a water-immersed objective set at ×40 (C-Apochromat/1.2 W corr) equipped with an Axioplan 2 imaging microscope (Carl Zeiss). The images were typically 644 × 130 pixels, corresponding to an image size of 75 × 15.1 μm using a pixel size of 0.12 μm. The images were collected every 0.5 μs, at intervals of 0.25 μs, for 2 min.

Data are expressed as means ± S.E.M. of n experiments. The results were analyzed using Student’s *t-*test and Mann-Whitney *U*-test.

## 4. Conclusions

In conclusion, we have established a system which allows prompt and accurate analysis of axonal transport that can visualize moving particles, and which may be applied together with other probes and recent imaging techniques. Future studies may include application of this assay system to analyze frequency of movement measured, distance traveled and other complex patterns of movement per cargo particle. This system affords a chemical biology strategy for evaluating neurotoxic effects of candidate therapeutic agents and chemicals for side-effect, hazard and risk assessment, and for developing novel therapeutic agents to restore affected axonal transport in various kinds of neuronal cells originating from central and peripheral nervous tissue, or disease-related pluripotent stem cells [42].
